# Peripheral T cell lymphoma, not otherwise specified: the stuff of genes, dreams and therapies

**DOI:** 10.1136/jcp.2008.055335

**Published:** 2008-08-28

**Authors:** C Agostinelli, P P Piccaluga, P Went, M Rossi, A Gazzola, S Righi, T Sista, C Campidelli, P L Zinzani, B Falini, S A Pileri

**Affiliations:** 1 Department of Haematology and Clinical Oncology “L and A Seràgnoli”, Bologna University School of Medicine, Bologna, Italy; 2 Institute of Pathology, Triemli Hospital, Zurich, Switzerland; 3 Institute of Haematology, Perugia University School of Medicine, Perugia, Italy

## Abstract

Peripheral T cell lymphomas (PTCL) account for about 12% of lymphoid tumours worldwide. Almost half show such morphological and molecular variability as to hamper any further classification, and to justify their inclusion in a waste-basket category termed “not otherwise specified (NOS)”. The latter term is used for neoplasms with aggressive presentation, poor response to therapy and dismal prognosis. In contrast to B cell lymphomas, PTCL have been the subject of only a limited number of studies to elucidate their pathobiology and identify novel pharmacological approaches. Herewith, the authors revise the most recent contributions on the subject based on the experience they have gained in the extensive application of microarray technologies. PTCL/NOS are characterised by erratic expression of T cell associated antigens, including CD4 and CD52, which have recently been proposed as targets for ad hoc immunotherapies. PTCL/NOS also show variable Ki-67 marking, with rates >80% heralding a worse prognosis. Gene expression profiling studies have revealed that PTCL/NOS derive from activated T lymphocytes, more often of the CD4+ type, and bear a signature composed of 155 genes and related products that play a pivotal role in cell signalling transduction, proliferation, apoptosis and matrix remodelling. This observation seems to pave the way for the use of innovative drugs such as tyrosine kinase and histone deacetylase inhibitors whose efficacy has been proven in PTCL primary cell cultures. Gene expression profiling also allows better distinction of PTCL/NOS from angioimmunoblastic T cell lymphoma, the latter being characterised by follicular T helper lymphocyte derivation and CXCL13, PD1 and vascular endothelial growth factor expression.

Peripheral T cell lymphomas (PTCL) represent approximately 12% of lymphoid neoplasms.^1^ Their incidence varies among countries, and it is higher in human T-cell lymphotropic virus-1 endemic areas.^1^ PTCL are a heterogeneous group of tumours that can be roughly subdivided into: specified and not otherwise specified (NOS) (Box 1).^1^ ^2^ While specified tumours correspond to distinct but rare entities often occurring at extranodal sites, NOS represent the commonest type of TCL (40–50%), followed by the angioimmunoblastic (AITL) and the anaplastic large cell (ALCL) types.

**Box 1: Mature T cell and NK cell neoplasms^1^**

**Peripheral T cell lymphoma, not otherwise specified (PTCL/NOS)**

**Peripheral T cell lymphoma, specified**

Leukaemic:

- T cell prolymphocytic leukaemia
- T cell large granular lymphocytic leukaemia
- Aggressive NK cell leukaemia
- Systemic Epstein–Barr virus positive T cell lymphoproliferative disease of childhood (associated with chronic active EBV infection)
- Hydroa vaccineforme-like lymphoma
- Adult T cell leukaemia/lymphoma

Extranodal:

- Extranodal NK/T cell lymphoma, nasal type
- Enteropathy-associated T cell lymphoma
- Hepatosplenic T cell lymphoma
- Subcutaneous panniculitis-like T cell lymphoma
- Mycosis fungoides
- Sézary syndrome
- Primary cutaneous anaplastic large-cell lymphoma
- Primary cutaneous aggressive epidermotropic CD8+ cytotoxic T cell lymphoma (provisional entity)
- Primary cutaneous γδ T cell lymphoma
- Primary cutaneous small/medium CD4+ T cell lymphoma (provisional entity)

Prevalently nodal:

- Angioimmunoblastic T cell lymphoma
- Anaplastic large cell lymphoma (ALCL), anaplastic large cell lymphoma kinase (ALK) positive
- ALCL, ALK negative (provisional entity)

PTCL/NOS cannot be further classified based on morphology, phenotype and molecular biology in most instances,^3^^–^5 although rare distinctive variants have been reported (ie, follicular and lymphoepithelioid).^6^^–^8 Usually, PTCL/NOS occurs in the fifth to sixth decade of life, and there is no evidence of sex predilection.^4^ ^9^ ^10^ PTCL/NOS more often presents in stage III–IV, with nodal, skin, liver, spleen, bone-marrow or peripheral blood involvement.^4^ ^9^ ^10^

The tumour is highly variable in terms of cell morphology and may contain prominent reactive components.^1^ ^3^

Immunohistochemistry usually shows T cell associated molecule expression, although the phenotypic profile is aberrant in about 80% of cases.^1^ ^3^

Clonal rearrangements of T cell receptor encoding genes are generally detected.^11^ The karyotype is aberrant in most cases, and is often characterised by complex abnormalities.^12^ Recently, recurrent chromosomal gains and losses have been documented in PTCL/NOS by comparative genomic hybridisation, and these have been found to differ from those seen in AITL and ALCL.^12^ ^13^

The molecular pathobiology of PTCL/NOS, as in general in all T cell neoplasms, is poorly understood. In particular, only limited numbers of studies have explored the gene expression profile (GEP).^14^^–^22

On clinical grounds, PTCL/NOS are among the most aggressive non-Hodgkin lymphomas. Their response to conventional chemotherapy is indeed poor, with 5-year relapse-free and overall survival rates of 26% and 20%, respectively.^4^ ^5^ ^9^ ^23^^–^26 Neither the morphology nor the international prognostic index (IPI) significantly correlates with the outcome. Clinical or clinicobiological scores have been proposed to identify cases with different prognoses.^26^ ^27^ However, the molecular bases of PTCL/NOS drug resistance and aggressiveness remain elusive.

In the following, the results recently obtained by our group through the extensive application of microarray technologies will be summarised and commented on, with the scope of defining the pathobiological characteristics of PTCL/NOS, tracing the borders between it and AITL on the one hand and anaplastic large cell lymphoma kinase (ALK)-negative ALCL on the other, and drawing attention to potentially novel prognosticators and therapeutic targets.^19^^–^22 27

## PHENOTYPIC PROFILE OF PTCL/NOS

**Figure 1:**
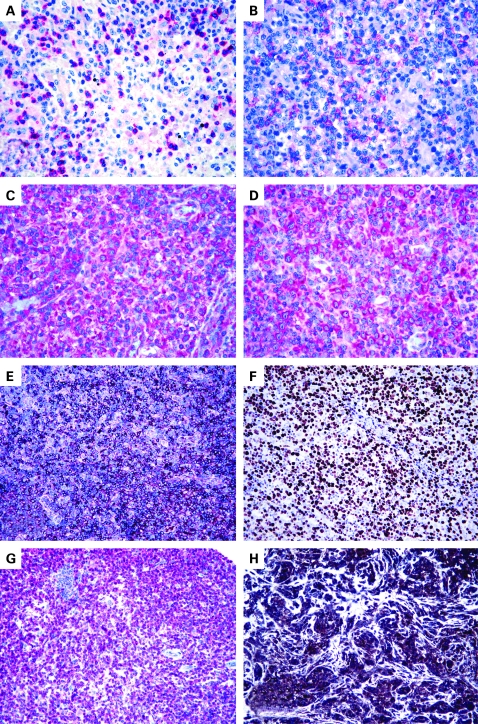
(A) Lymphomatous cells do not express CD4; however, CD4 is detected in some reactive small lymphocytes (alkaline phosphatase anti-alkaline phosphatase (APAAP) technique, Gill’s haematoxylin nuclear counterstaining, ×250). (B) Partial CD30 expression; it should be noted that the tumour has no anaplastic morphology (APAAP technique, Gill’s haematoxylin nuclear counterstaining, ×250). (C) Positivity for platelet-derived growth factor receptor α (PDGFRα) (APAAP technique, Gill’s haematoxylin nuclear counterstaining, ×400). (D) PDGFRα is phosphorylated (APAAP technique, Gill’s haematoxylin nuclear counterstaining, ×400). (E) CXCL13 expression by neoplastic elements in angioimmunoblastic T cell (EnVision+ technique, Gill’s haematoxylin nuclear counterstaining, ×100). (F) Ki-67 marking exceeds the 80% value (EnVision+ technique, Gill’s haematoxylin nuclear counterstaining, ×200). (G) CD52 positivity in a peripheral T cell lymphoma, not otherwise specified (APAAP technique, Gill’s haematoxylin nuclear counterstaining, ×100). (H) Strong expression of vascular endothelial growth factor in an angioimmunoblastic T cell lymphoma (EnVision+ technique, Gill’s haematoxylin nuclear counterstaining, ×200).

As mentioned above, PTCL/NOS usually carry phenotypic aberrations, the exact prevalence and spectrum of which have remained unresolved.^8^ ^11^ ^25^ ^28^ In 2006, we reported PTCL from 193 Italian patients (148 NOS and 45 AITL) that had been collected on tissue microarrays and tested by immunohistochemistry and Epstein–Barr virus encoded RNA 1 (EBER1) and EBER2 in situ hybridisation.^27^ The βF1 antibody (raised against the T cell receptor β chain) reacted with 96% of tumours. NOS and AITL PTCL demonstrated frequent loss of CD5 and CD7, with CD3 being the conventional marker most commonly expressed in NOS types, and CD2 in the AITL types. CD4 was detected in 46% of cases (see fig 1A) and CD8 in 15% of cases; these results are in line with those reported in previous publications.^8^ ^11^ ^25^ ^28^ ^29^ Interestingly, we found 32% of AITLs to be CD8+; this is in the upper range of reported values.^27^ ^30^^–^44 In contrast, the incidence of CD4 positivity (42%) was much lower than expected.^27^ ^45^ Interestingly, a huge number of PTCL/NOS and AITL (55%) turned out to be either CD4/CD8 double-negative or, more rarely, double-positive. Such profiles, which are usually observed during intrathymic T cell development,^1^ ^27^ had previously been reported in isolated PTCL cases^46^ ^47^ and a proportion of cutaneous T cell tumours.^27^ ^48^ Furthermore, CD10 expression was detected in only 39% of AITL, even when adopting a low cut-off value.^27^ Such rates did not vary between tissue microarrays and conventional sections.

CD56 was detected in 5% of PTCL/NOS: all cases stained with βF1 and three co-expressed TIA-1. Interestingly, CD56 expression suggests a malignant phenotype: in fact, under physiological conditions it is limited to T lymphocytes with spontaneous non-MHC-restricted cytotoxicity.^27^ ^49^ CD57 was seen in 10% and 5% of PTCL/NOS and AITL respectively. Although numbers of CD57+ normal T lymphocytes increase with age,^49^ no correlation was found between patient age and CD57 expression.^27^ ^50^

CD30 was recorded in 6% of cases (see fig 1B), CD15 in 4%, and CD20 in 1%^27^; these rates of positivity may undoubtedly cause diagnostic difficulties. In particular, CD20 was detected in only two PTCL/NOS that were negative for CD79a, in keeping with previous observations of CD20 positivity in isolated PTCL/NOS, and CD79a aberrant expression in “specified” PTCL.^27^ ^51^^–^53 Co-expression of CD15 and CD30 was found in only 3/183 of cases that were able to be evaluated. This is the first reliable estimate of the random incidence of such a phenomenon in a large cohort of patients with PTCL; in fact, the previous reports of Barry *et al*^54^ and Gorczyka *et al*^55^ referred to a highly selected series. In spite of its rarity, such a finding raises the question of how to differentiate between PTCL and classic Hodgkin lymphoma (CHL) under these circumstances: the polymorphism of neoplastic elements, the possible lack of Reed-Sternberg cells and B cell specific activator protein negativity favour the diagnosis of PTCL and vice versa. In particular, B cell specific activator protein is a valuable B cell marker that is found in about 90% of cases of CHL,^56^ but it is exceptional in PTCL/NOS.^57^

In our hands, the mean percentage of Ki-67+ neoplastic cells was around 50%, with 11% of PTCL/NOS exceeding the 80% value. Finally, EBV integration was found at the neoplastic cell level in 5% and 3% of PTCL/NOS and AITL respectively; this value is definitely lower than the one recorded by Dupuis *et al* in a French cohort.^58^

## GEP OF PTCL/NOS

PTCL have been the subject of a limited number of GEP studies^14^^–^22 59 60 (table 1). In particular, Tracey *et al*,^60^ Lamant *et al*^16^ and de Leval *et al*^17^ focused on mycosis fungoides, ALK-positive and -negative ALCLs, and AITL, respectively. In contrast, Martinez-Delgado *et al*^14^ and Ballester *et al*^15^ analysed large collections of PTCL of the NOS, AITL and ALCL types. However, their studies suffered limitations that varied from the usage of chips with a restricted number of genes^14^ ^15^ to the lack of a reliable normal counterpart for comparison.^14^ Martinez-Delgado *et al*^14^ reported that PTCL/NOS corresponded to a heterogeneous group of tumours whose GEP was difficult to interpret due to the amount of infiltrating reactive cells. According to those authors, the only clinically relevant information provided by GEP pertains the NF-κB gene expression level (see below).^14^ Ballester *et al*^15^ reported that GEP could discriminate among PTCL of the NOS, AITL and ALCL types, although NOS did not share a single profile. Using a multiclass predictor, the authors separated their cases into three molecular subgroups: U1, U2 and U3. However, the corresponding signatures might have been, at least in part, influenced by reactive components, as suggested by the fact that, for instance, the U3 subgroup consisted almost entirely of histiocyte-rich tumours.

**Table 1:** The main studies dealing with gene expression profiling of peripheral T cell lymphomas

Reference	Disease(s) explored	Comments
Tracey *et al*^60^	FM	The GEP of FM was investigated, and it showed concurrent deregulation of multiple genes involved in the tumour necrosis factor signalling pathway.
Martinez-Delgado *et al*^14^	PTCL/NOS	The authors found significant differences between the peripheral and lymphoblastic T cell lymphomas. The differences included a deregulation of the nuclear factor-κB signalling pathway.
Martinez-Delgado *et al*^98^	PTCL/NOS	The authors found two different subgroups of PTCL based on the expression of NF-κB related genes. One-third of PTCL clearly showed reduced expression of NF-κB genes, while the other group was characterised by high expression of these genes. Of interest, the expression profile associated with reduced expression of NF-κB genes was significantly associated with shorter survival of patients.
Ballester *et al*^15^	PTCL/NOS, AILT, ALCL	According to this study, PTCL/NOS could be divided into three molecular subgroups: U1, U2 and U3. The *U1* gene expression signature included genes known to be associated with poor outcome in other tumours, such as *CCND2*. The U2 subgroup was associated with overexpression of genes involved in T cell activation and apoptosis, including NF-κB1 and BCL-2. The U3 subgroup was mainly defined by overexpression of genes involved in the IFN/JAK/STAT pathway. Notably, such distinction possibly reflected, at least in part, the presence of reactive components in the PTCL samples.
de Leval *et al*^17^	AILT	The molecular profile of AILT was characterised by a strong microenvironment and overexpression of several genes characteristic of normal follicular helper T (TFH) cells: *CXCL13, BCL6*, *PDCD1*, *CD40L* and *NFATC1*. Such a finding was reinforced by gene set enrichment analysis, which demonstrated that the AITL molecular signature was significantly enriched in TFH-specific genes.
Piccaluga *et al*^20^	PTCL/NOS	The authors showed that PTCL/NOS are most closely related to activated peripheral T lymphocytes, either CD4+ or CD8+, based on the GEP. In addition, PTCL/NOS displayed deregulation of relevant functional cell programmes. In particular, among others, *PDGFRA*, a gene encoding for a tyrosine kinase receptor, turned out to be aberrantly expressed by PTCL/NOS. Notably, phosphorylation of PDGFRA and sensitivity of cultured PTCL cells to imatinib were demonstrated.
Piccaluga *et al*^21^	PTCL/NOS	The authors found that CD52 is expressed in approximately 40% of PTCL/NOS at the same level as in normal T lymphocytes, being aberrantly downregulated in the remaining cases. Notably, they concluded that the estimation of CD52 expression may provide a rationale for the selection of patients with a higher probability of response to the anti-CD52 antibody alemtuzumab.
Piccaluga *et al*^22^	AILT	In this manuscript, the authors reported that AILT and other PTCL have rather similar GEP, possibly sharing common oncogenic pathways. In addition, they found that the molecular signature of follicular T helper cells was significantly overexpressed in AILT. Finally, several genes, such as *PDGFRA* and *VEGF*, which are deregulated in AILT and represent potential therapeutic targets, were identified.
Lamant *et al*^16^	ALCL	This was the first study to focus on ALCL. Unsupervised analysis classified ALCL in two clusters, corresponding essentially to morphological subgroups and clinical variables. Supervised analysis showed that ALK-positive ALCL and ALK-negative ALCL have different GEP, further confirming that they are different entities.
Cuadros *et al*^18^	PTCL/NOS	Five clusters of genes were identified, and their expression varied significantly among the samples. Genes in these clusters were functionally related to different cellular processes such as proliferation, inflammatory response, and T cell or B cell lineages. Notably, overexpression of genes in the proliferation signature was significantly associated with shorter survival of patients.

AILT, peripheral T cell lymphoma, angioimmunoblastic type; ALCL, anaplastic large cell lymphoma; ALK, anaplastic large cell lymphoma kinase; FM, mycosis fungoides; GEP, gene expression profile; PDGFRA, platelet-derived growth factor receptor α; PTCL/NOS, peripheral T cell lymphoma, not otherwise specified.

Recently, we^20^ published a GEP study based on the analysis of 28 PTCL/NOS, all corresponding to lymph node biopsy samples containing an amount of neoplastic cells exceeding 70% value of the whole examined population. The mRNA extracted from these cases was hybridised on the HG U133 2.0 Plus gene chip. The results obtained were compared with those of six AITL, six ALCL (two ALK-positive and four ALK-negative) and 20 samples of normal T lymphocytes, which were purified from the peripheral blood and tonsil and corresponded to the main T cell subsets (CD4+, CD8+, resting and activated). Such a study significantly differed from most previous reports^14^ ^60^ in terms of methodology and selection criteria. In addition, for the first time it provided the rationale for possible targeted therapies in PTCL/NOS by offering clear evidence of their ex vivo effectiveness.

In particular, the GEP we detected^20^ indicated that PTCL/NOS are distinct from normal T and B lymphocytes and are more closely related to activated rather than resting T cells. As in normal mature T lymphocytes, it was possible to identify two main subgroups of PTCL/NOS, with GEPs related to either CD4 or CD8 elements. Notably, this characteristic did not reflect the expression of CD4 and CD8 molecules.

In addition to histogenetic information, our analysis^20^ provided several insights into the functional alterations of PTCL/NOS. A careful comparison of PTCL/NOS with the closest normal counterparts revealed the systematic deregulation of 155 genes controlling functions that are typically damaged in malignant cells, such as matrix remodelling, cell adhesion, transcription, proliferation and apoptosis. In particular, our findings might explain the dissemination pattern of PTCL/NOS, with frequent extranodal and bone-marrow involvement and spread to peripheral blood,^1^ by showing the upregulation of *FN1*, *LAMB1*, *COL1A2*, *COL3A1*, *COL4A1*, *COL4A2*, and *COL12A1* (ie, genes that promote local invasion and metastasis in different types of human cancer).^61^^–^63 In addition, it revealed the deregulation of genes involved in apoptosis (eg, *MOAP1*, *ING3*, *GADD45A* and *GADD45B*)^64^^–^70 and chemoresistance (such as *CYR61* and *NNMT*).^61^^–^63,71^–^82

Immunohistochemistry provided in situ validation of the genomic data by showing correspondence between mRNA and protein expression, as seen, for example, with GEP, *PDGFRα* (see fig 1C and D) and *BCL10*. In addition, by comparison with normal tissues, immunohistochemistry allowed the identification of staining patterns corresponding to the synthesis of ectopic or paraphysiological products by neoplastic cells. Finally, the phenotypic test highlighted the possibility that some of the results obtained by GEP may depend on non-neoplastic components present in the analysed sample, as seen for Caldesmon.

In the course of the same study, we found that all ALCLs tended to cluster together – irrespective of their ALK positivity or negativity – showing a signature distinct from those of PTCL/NOS and AITL.^20^

**Figure 2:**
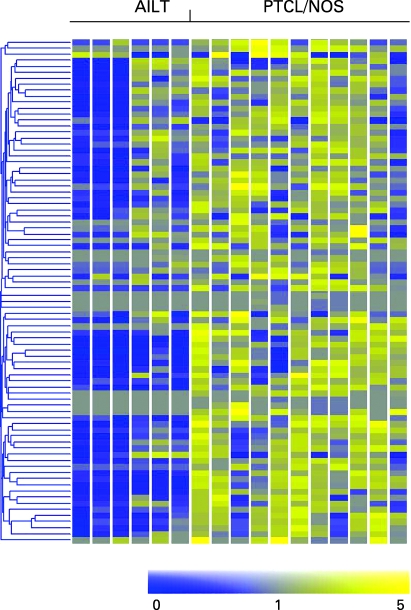
Peripheral T cell lymphoma, not otherwise specified (PTCL/NOS), and peripheral T cell lymphoma, angioimmunoblastic type (AILT), can be distinguished according to their gene expression profile. Eighty-three differentially expressed genes are plotted in the matrix.

More recently, we succeeded in identifying a gene signature discriminating between PTCL/NOS and AITL (fig 2).^22^ In addition, the observed AITL global profile strengthened its derivation from the follicular T helper lymphocyte (FT_H_L), as originally proposed by Rüdiger *et al*^83^ and de Leval *et al*.^17^ Among upregulated genes, were those encoding for CXC13, PD1 and vascular endothelial growth factor (VEGF).

## PRACTICAL IMPLICATIONS OF PHENOTYPIC AND MOLECULAR FINDINGS

### Diagnosis

Along with clonality studies,^11^ the phenotype plays a basic role in the distinction of PTCL from reactive conditions—such as paracortex hyperplasia—that can mimic malignant lymphoma. In fact, the lack of one or more T cell associated antigens (see above) is a hallmark of neoplastic cells as opposed to the complete phenotype of normal T lymphocytes.^27^ Immunohistochemical and molecular findings are also of great value for differential diagnosis among PTCL.

#### PTCL/NOS versus AITL

Such distinction may be problematic in about 25% of cases, based on conventional criteria.^84^ Also CD10 staining, proposed as characteristic of AITL,^85^ ^86^ is actually seen in less than 50% of cases in our experience.^27^

Notably, the AITL gene signature recently reported by de Leval *et al*^17^ and our group^22^ (see above) provides a rationale to the immunohistochemical observations of Dupuis *et al*,^87^ Grogg *et al*^88^ and Roncador *et al*^89^ who found that most, if not all, AITL stain for typical FT_H_L-related antigens, such as CXCL13 (see fig 1E) and PD-1. Such molecules can actually represent a powerful tool for the distinction of AITL from PTCL/NOS, due to the exceptional positivity of the latter, a finding also confirmed in our PTCL tissue microarray (unpublished observation).

#### PTCL/NOS versus ALCL

Lamant *et al*^16^ reported that ALK-positive and ALK-negative ALCL have different GEPs. In particular, they found that *BCL-6*, *PTPN12*, *C/EBPβ* and *serpinA1* genes overexpressed in ALK-positive ALCL, a result also confirmed at the protein level. In contrast, the molecular signature of ALK-negative ALCL included overexpression of *CCR7*, *CNTFR*, *IL22* and *IL21* genes, but did not provide any obvious clues to its molecular pathogenesis. This led to the question of whether ALK-negative ALCL should be included in PTCL/NOS. In the course of our GEP study, we found that all ALCL tended to cluster together irrespective of their ALK status, and this signature was clearly distinct from that of PTCL/NOS.^20^ In addition to suggesting that ALK-positive and ALK-negative ALCL probably share a set of deregulated pathways, our findings did not support the proposal that ALK-negative ALCL is a subtype of PTCL/NOS. Such a viewpoint is strengthened by the results of a recent clinicopathological trial showing that ALK-negative ALCL—although more aggressive than ALK-positive ALCL—has 5-year failure-free and overall survival rates that are significantly better than PTCL/NOS.^84^

### Prognosis

#### EBV, CD15 and proliferation

In our series of Italian patients, we found that high Ki-67 expression (see fig 1F), EBV status and CD15 staining were associated with the worst outcome in PTCL/NOS.^27^ Interestingly, a proliferation signature has recently been reported to correlate with an aggressive clinical course,^18^ and EBV has repeatedly been proposed as a negative prognosticator in PTCL.^58^ ^90^ ^91^ No other phenotypic marker alone or in combination was associated with a poor outcome, although patients with tumours expressing a CD57 or CD4+/CD8− profile showed a tendency towards a more favourable outcome, as also observed by others.^25^ ^48^

#### Clinicopathological score

Based on our collective results and those published in the literature,^26^ ^58^ ^92^^–^96 we developed a new score that integrates patient- and tumour-specific characteristics (age ⩾60 years, performance status, lactate dehydrogenase, and Ki-67 marking >80%) and identifies three clear-cut groups of patients with different prognosis. Such a score seems to be more effective than previous indices, including international prognostic index and prognostic index for peripheral T cell lymphoma, not otherwise specified.^26^

#### CYP3A

Recently, Rodríguez-Antona *et al*^97^ measured tumour *CYP3A* mRNA content in 44 T cell lymphomas and found a large variation in its expression that might be due to gains affecting the corresponding gene. To test whether *CYP3A* could influence PTCL treatment outcome, its expression levels were compared with the patient clinical response and survival, and it was observed that a high *CYP3A4* expression was significantly associated with a lower complete remission rate. These results indicate that *CYP3A* as a potential predictor of tumour chemosensitivity.

#### NF-κB pathway

Different GEP studies have suggested that PTCL/NOS may show up- or downregulation of NF-κB molecules,^14^ ^15^ ^98^ with possible prognostic implications (see above).^14^ ^98^ However, these studies included a limited number of PTCL/NOS^14^ or cases with prominent non-neoplastic components.^15^ By contrast, we found that PTCL/NOS mostly consisting of neoplastic cells present with global downregulation of NF-κB genes in comparison with normal T lymphocytes. This observation was corroborated by consistent cytoplasmic localisation of NF-κB molecules, the latter moving to the nucleus in the case of NF-κB pathway activation (unpublished observation).

### Therapy

#### CD4 and CD52 expression

The in vivo administration of monoclonal antibodies targeted to CD4 and CD52 has recently been proposed for the treatment of patients with PTCL.^99^ However, in our experience this should be regarded with caution when referring to PTCL/NOS. The latter, in fact, characteristically lacks the expression of one or more T cell associated antigens, including those antigens that these antibodies are targeted towards. In particular, we found that CD4 is lacking at the neoplastic cell level in up to 50% of cases.^27^ CD52 is a molecule expressed by most peripheral blood lymphocytes, macrophages, and monocytes.^102^ Campath-1H (alemtuzumab) is a humanised antibody against CD52 currently approved for B cell chronic lymphocytic leukaemia therapy,^103^^–^106 and it has also shown interesting activity in T prolymphocytic leukaemia and cutaneous TCLs.^107^ Although other factors can affect its response in vivo, the lack of CD52 expression may play a major role in causing refractoriness to the compound. Few data are available regarding the use alemtuzumab in PTCL/NOS.^108^ ^109^ We studied the expression of CD52 on tissue microarrays containing 97 PTCL/NOS.^21^ In addition, in 28 cases for which frozen material was available, GEP were generated and compared with those of 20 samples of normal T lymphocytes.^21^ We found that 17/28 (60%) PTCL/NOS showed CD52 gene expression level lower than the lowest one recorded in normal T cells.^21^ In addition, the gene product was detected by immunohistochemistry in 40/97 (41%) PTCL (see fig 1G).^21^ Interestingly, such data are in keeping with the clinical results obtained by Enblad *et al*^108^ who found an overall response rate of 36% in PTCL treated with alemtuzumab. Based on these findings, we think that the estimation of CD52 expression may provide a rationale for the selection of patients with higher probability of responding to alemtuzumab, by avoiding the risk of unwanted toxicity.^21^ Similar conclusions were achieved by Rodig *et al*^100^ and Chang *et al*,^101^ who reported immunohistochemical detection of CD52 in 0–40% of PTCL.

#### PDGFRα

The regular detection of PDGFRα overexpression at the mRNA and protein levels, as well as its frequent phosphorylation (see fig 1D), prompted us^20^ to design an ex vivo experiment aimed testing the sensitivity of PTCL/NOS cells to imatinib, a well-known PDGFRα inhibitor.^110^ The results obtained were of interest, with about 50% cytotoxic effect seen at 48 h with a 1 μmol concentration. Such an effect became even higher (75%) with a 10 μmol dose. Notably, imatinib exerted a limited effect on the viability of normal lymphocytes.

**Take-home messages**

- Peripheral T cell lymphomas (PTCL) represent about 12% of all lymphoid tumours worldwide. Around half belong to the not otherwise specified (NOS) type.
- Conventional morphological and molecular criteria do not assist in the subclassification of PTCL/NOS, as anthracycline-based therapies fail to cure it, and most patients die of their disease within 5 years.
- Novel microarray technologies allow the identification of peculiar features that may in turn be useful for the diagnosis, prognosis and treatment of PTCL/NOS.
- PTCL/NOS is characterised by frequent defective expression of T associated antigens, including CD4 and CD52, which have recently been proposed as targets for humanised monoclonal antibodies.
- The growth fraction >80% has a prognostic impact.
- Gene expression profiling studies show derivation from activated peripheral T lymphocytes and systematic deregulation of 155 genes and related products that may provide the rationale for the unprecedented usage of drugs such as tyrosine kinase and histone deacetylase inhibitors.
- The gene expression profile also contributes to the better definition of the boundaries between PTCL/NOS and angioimmunoblastic T cell lymphoma, the latter deriving from follicular T helper lymphocytes and characteristically expressing CXCL13 and PD1 along with vascular endothelial growth factor.

#### Histone deacetylation

Since silencing of certain genes (such as *GADD45A* and *GADD45B*) can be regulated by epigenetic mechanisms including acetylation, we tested a histone deacetylase inhibitor (HDACi) (ITF2357) against PTCL/NOS primary cells. Notably, the compound induced dramatic G0–G1 cell cycle arrest and apoptosis at therapeutic concentrations, suggesting a possible role for this class of drugs in PTCL/NOS therapy, as also supported by preliminary clinical observations.^111^ Interestingly, the combination of ITF2357 and daunorubicin apparently had a slight additive effect, as already observed with other HDACi.^112^

#### VEGF

Recently, we observed upregulation of the *VEGF* gene in AITL.^22^ The same finding had previously been reported by de Leval *et al*^17^ who had attributed it to the rich vascular component of the tumour. However, by immunohistochemistry on tissue microarrays, we showed that neoplastic cells strongly express both VEGF (see fig 1H) and its receptor KDR.^22^ This fact suggests possible AITL sensitivity to anti-angiogenetic drugs, such as thalidomide and bevacizumab.^113^

## CONCLUSIONS

For a long time, PTCL have represented an orphan pathology. This can be explained by their relatively low prevalence (which is in any case higher than that of a “common” tumour, such as CHL), diagnostic difficulties and dismal prognosis. Based on recent advances in the genomic and translational fields, a new scenario can now be envisaged leading the way to more successful therapeutic strategies. This may be the right time to live a dream, never forgetting however that “the truth is not always pure and never simple” (Oscar Wilde).
